# Therapeutic Properties of Antipyrine

**Published:** 1887-03

**Authors:** 


### THERAPEUTIC PROPERTIES OF ANTIPYRINE.

Dr. Jno. Blake pointed out (*N. Y. Medical Record*) in September 1886, that antipyrine possesses a remarkable power in controlling

*headache* from whatever cause arising—whether from indigestion, loss of sleep, menstrual disturbance, or mental fatigue; and that it is also efficacious in uræmic headache and recurrent attacks of cranial neuralgia; it also acted as a prophylactic in sick headache. The *N. Y. Medical Record* says these observations received independent confirmation nearly at the same time by Ungar in Germany. Ungar (Nov. '86) obtained excellent results in hemicrania. It has been used in Germany successfully as a dressing to indolent ulcers; the surface of the ulcer is covered with the dressing powder and a layer of salicylated cotton. This was replaced with iodoform after granulation had become established. Dr. Casati (*Medical Record*) found antipyrine a valuable hæmostatic; and Lavarand succeeded in arresting a nose-bleed by means of a three per cent solution on lint introduced far up into the nasal cavity, where everything else failed, even to plugging. It is said to be prompt and certain in its action, and even superior to perchloride of iron. In the *Manual of Medicine and Surgery* a comparison is made between antipyrine, salicylic acid, and quinine as antipyritics, and it is claimed that there is less danger from cardiac depression following antipyrine than from either of the others. It is quite unfortunate that this valuable remedy should be a "patent" medicine, for it promises to become a "standby" in many diseases. Its use in France is prohibited by "patent" laws.

